# Characterization of Dedifferentiating Human Mature Adipocytes from the Visceral and Subcutaneous Fat Compartments: Fibroblast-Activation Protein Alpha and Dipeptidyl Peptidase 4 as Major Components of Matrix Remodeling

**DOI:** 10.1371/journal.pone.0122065

**Published:** 2015-03-27

**Authors:** Julie Lessard, Mélissa Pelletier, Laurent Biertho, Simon Biron, Simon Marceau, Frédéric-Simon Hould, Stéfane Lebel, Fady Moustarah, Odette Lescelleur, Picard Marceau, André Tchernof

**Affiliations:** 1 Centre de Recherche de l’Institut Universitaire de Cardiologie et de Pneumologie de Québec, Quebec City, QC, Canada; 2 CHU de Québec Research Center, Quebec City, QC, Canada; 3 Department of Nutrition, Laval University, Quebec City, Quebec, Canada; University of Minnesota—Twin Cities, UNITED STATES

## Abstract

Mature adipocytes can reverse their phenotype to become fibroblast-like cells. This is achieved by ceiling culture and the resulting cells, called dedifferentiated fat (DFAT) cells, are multipotent. Beyond the potential value of these cells for regenerative medicine, the dedifferentiation process itself raises many questions about cellular plasticity and the pathways implicated in cell behavior. This work has been performed with the objective of obtaining new information on adipocyte dedifferentiation, especially pertaining to new targets that may be involved in cellular fate changes. To do so, omental and subcutaneous mature adipocytes sampled from severely obese subjects have been dedifferentiated by ceiling culture. An experimental design with various time points along the dedifferentiation process has been utilized to better understand this process. Cell size, gene and protein expression as well as cytokine secretion were investigated. Il-6, IL-8, SerpinE1 and VEGF secretion were increased during dedifferentiation, whereas MIF-1 secretion was transiently increased. A marked decrease in expression of mature adipocyte transcripts (PPARγ2, C/EBPα, LPL and Adiponectin) was detected early in the process. In addition, some matrix remodeling transcripts (FAP, DPP4, MMP1 and TGFβ1) were rapidly and strongly up-regulated. FAP and DPP4 proteins were simultaneously induced in dedifferentiating mature adipocytes supporting a potential role for these enzymes in adipose tissue remodeling and cell plasticity.

## Introduction

Adipose tissue is composed of several cell types including preadipocytes, mature adipocytes, endothelial and immune cells [1]. During excess caloric intake, adipocyte differentiation occurs through a process termed adipogenesis during which mesenchymal stem cells committed into preadipocytes become mature adipocytes, i.e. cells specialized in lipid storage [2]. This process has been considered as a terminal event for many years. However, although transdifferentiation is still controversial in the literature, recent publications have revealed that mature white adipocytes may dedifferentiate into precursor cells [3] or, transdifferentiate into other cell types such as brite/beige adipocytes [4,5]. Dedifferentiation is achieved *in vitro* by a technique called ceiling culture, during which the standard culture flask is completely filled with medium and is incubated upside down [6]. This technique allows floating mature adipocytes to adhere to the treated face of the tissue culture flask. After approximately a week, most cells acquire a fibroblast-like phenotype.

Ceiling culture has been performed with cells from mice, rats, pigs, and more recently with human adipocytes and generated dedifferentiated fat (DFAT) cells from all these species [3,7–10]. DFAT cells express embryonic stem cell markers such as Oct4 and Nanog [8] and are closely related to bone-marrow stem cells (BMSC) based on similarities in surface antigens and epigenetic signatures [8,11]. As multipotent cells, they have the capacity to re-differentiate into adipogenic, osteogenic and chondrogenic lineages [3,8]. Moreover, Poloni et al. also produced neurospheres indicating that their multipotency may go beyond mesodermic lineages [8]. The potential of DFAT cells for regenerative medicine is significant, considering the abundance of cells generated from a small piece of adipose tissue, the purity of the resulting population, their long-term replicative capacity as fibroblast-like cells and their multipotency [12].

Besides the potential of DFAT cells to act as stem cells, little is known about the dedifferentiation process itself. Cells undergo a complex morphological change that most likely involves dramatic modification of their cellular programs. Using porcine adipocytes, Ono et al. identified some programs that are up-regulated (e.g.: cell morphogenesis) or down-regulated (e.g.: lipid metabolism) in fully dedifferentiated fat cells compared to mature adipocytes [13]. The purpose of our study was to investigate the physiological process of mature adipocyte dedifferentiation by evaluating cytokine secretion as well as gene and protein expression at different time-points during the process. To do so, we dedifferentiated omental (OM) and subcutaneous (SC) mature adipocytes from obese men and women.

## Methods

### Tissue sampling and collagenase digestion

Adipose tissue samples were obtained from men and women undergoing bariatric surgery as treatment for severe obesity. The project was approved by the Research Ethics Committee of the Institut universitaire de cardiologie et de pneumologie de Québec (IUCPQ). Written and informed consent was obtained through the management framework of the IUCPQ Obesity Tissue Bank prior to tissue sampling. Samples were collected at the time of the surgery from two different abdominal fat depots: the greater omentum and the abdominal SC fat compartment. Fat samples were from men and women aged between 29 and 66 years with a mean body mass index of 51.5 kg/m^2^ (range: 35.5–71.8 kg/m^2^). Portions of adipose tissues were quickly frozen in liquid nitrogen or fixed in 10% formalin for paraffin embedding. The rest of the tissue was digested by collagenase as previously described by our group [14]. Isolated mature adipocytes were used for ceiling culture whereas the stromal vascular fraction was seeded in culture flasks. The culture conditions used [14] excluded the erythrocyte lysis buffer step and allowed the enrichment of preadipocytes that were then harvested with Qiazol reagent for RNA isolation.

### Ceiling culture

Isolated mature adipocytes were counted and 500 000 cells were added to a T-25 flask completely filled with DMEM-F12 supplemented with 20% calf serum [6]. Flasks were incubated upside down at 37°C, 5% CO_2_ for seven days, and then reversed to pursue standard culture until day 12 in the same medium. For gene expression analysis, OM and SC ceiling culture from each patient were harvested at day 4 and day 7. One flask per depot per patient was reversed at day 7 and maintained in culture for an additional 5 days (corresponding to day 12). Time points were chosen based on our observations that harvesting cells at day 4 provides a round cell population that has completely adhered to the flask. All ceiling cultures performed in the laboratory were reversed at day 7. Day 12 represents a time point where the majority of cells were fibroblast-like cells. When DFAT cells were necessary for other purposes, cultures were maintained in standard condition for more than 12 days and passed when cells reached confluence. A ceiling culture model in 6-well plates was elaborated. Eight ml of DMEM/F12-20% calf serum was added to the well containing a ½” plastic bushing (Iberville, Memphis, TN) supporting a glass slide. A second glass slide was put on the bushing and mature adipocytes were seeded under the coverslip. Cells floated and adhered to the slides and at specific time points, were fixed in 10% formalin for future analysis such as Oil Red O (ORO) staining, microscopy and computer imaging.

### Oil Red O Staining and confocal imaging

ORO staining was performed as previously described by Koopman and collaborators [15]. Briefly, cells fixed with 10% formalin were incubated in Oil Red O working solution for 30 minutes. After 3 washes, cells were counterstained using Mayer’s haematoxylin for 60 sec. Slides were rinsed and covered with coverslips using 10% glycerol in PBS. Images were immediately taken with confocal microscope Olympus BX51. Red pixel measurement was performed with Image J software (n = 6, 4M:2F). In our efforts to characterize the dedifferentiation process, we observed that red pixel measurement per surface provided indirect indication of process efficiency as the lipid droplets stained by ORO decreased in size during dedifferentiation.

### Cytokine detection

Human cytokine arrays panel A (36 cytokines-Cat. No ARY005) were used according to the manufacturer (R&D Systems) using 700 μl of media from 6-well OM and SC ceiling cultures at day 7 and 12 (n = 3). Human 27-plex was performed according to the Biorad protocol using media samples from OM and SC ceiling cultures at day 7 and 12 (n = 4, except for IL-8 day 7 where one sample value was above detection limit).

### RNA extraction and Real-time quantitative RT-PCR

RNA extraction was done using the QIAGEN RNeasy extraction kit. RNA was extracted from dedifferentiation time course experiments at day 0, day 4 and day 7 of ceiling culture and at day 12, from 4 subjects for OM and SC cells. Subjects’ mean age was 44.5 years (min-max: 35–59) and mean BMI was 44.35 (min-max: 39–52). For each sample, cDNA was obtained using the Quantitect reverse transcriptase kit (Qiagen). The following sequences were used for quantitative PCR (forward/reverse): ATP synthase, H+ transporting, mitochondrial F1 complex, O subunit (ATP5o): 5’-AACGACTCCTTGGGTATTGCTTAA-3’/5’-ATTGAAGGTCGCTATGC-CACAG-3’, Glucose-6-phosphate dehydrogenase (G6PD): 5’ GCAGGGCATTGAGGTTGG-GAG-3’/5’-GATGTCCCCTGTCCCACCAACTCTG3’, Peroxysome Proliferator-Activated Receptor gamma 2 (PPARγ2): 5’-TTGCAGACAGTGTATCAGTGAAGGAAT-3’/5’-ATTACAGCAAACCCCTATTCCA-TG-3’, CCAAT Enhanced Binding Protein alpha (C/EBPα): 5’-TTCACATTGCACAAGGCACT-3’/5’-GAGGGACCGGAGTTATGACA-3’, Lipoprotein Lipase (LPL): 5’-ATTCAGAGACTTG-TCATGGCATTTC-3’/5’-TCGCCATTCAGAAGATCAGAGTAAA-3’ Adiponectin (ADIPOQ): 5’-TAGAACAGCTCCCAGCAACA-3’/5’-CCATCTCCTCCTCACTTCCA-3’, Fibroblast Activation Protein alpha (FAP): 5’-TGTCCTGAAATCCAGTTTGG-3’/5’-GTGCATTGTCTTACGCCCTT -3’, Dipeptidyl Peptidase IV (DPP4): 5’-GCGACTGTCAGCTGTAGCAT-3’/5’-TGAAGACA-CCGTGGAAGGTT-3’, Matrix-Metalloproteinase 1 (MMP1): 5’-TTGTGGCCAGAAAACAGAAA-3’/5’-TTCGGGGAGAAGTGATGTTC-3’, Transforming Growth Factor β1 (TGFβ1): 5’-AAGTT-GGCATGGTAGCCCTT-3’/5’-CCCTGGACACCAACTATTGC-3’. Real-time RT-PCR was performed using SYBERGreen RT^2^ kit (QIAGEN) and a Biorad CFX96 C1000 Thermal Cycler. Housekeeping gene expression (ATP5o and G6PD) was measured in each sample. Results are presented as ΔΔCt relative to housekeeping gene expression. Graph bars represent average values of ΔΔCt values and error bars are the standard error means (SEM) calculated using the JMP software (SAS. Institute Inc). Both housekeeping genes yielded similar results, only G6PD results are shown.

### Western Blotting and antibodies

Proteins were extracted from the organic phase of the RNA phenol-chloroform extraction. First, 100% ethanol was added to the organic phase and incubated for 5 minutes. After centrifugation (4500 rpm, 2 minutes, 4°C), the surpernatant was incubated 10 minutes with 1.5 ml isopropanol. After centrifugation, the pellet was submitted to three washes with 1.5ml ethanol-0.3M guanidine with 20 minute incubation and to one additional wash without guanidine. The pellet was then incubated at 65°C in Tris pH 7.4–6% SDS until complete dissolution. Sonication was performed as a final disruption step. Protein samples were run on a 10% SDS-Page and transferred to nitrocellulose membrane. We used a mouse anti-FAP antibody (dilution 1/500) (Novus Biological), and a goat anti-DPP4 antibody (dilution 1/500) (R&D).

### Immunofluorescence

Ceiling culture was performed in 6-well plates using glass slides. Cells were fixed in 10% formalin. Washed, fixed cells were incubated in DAPI solution for 1 hour in the dark. Cells were then treated with TBS1x-0.2%Triton for 15 minutes. Primary antibodies (mouse anti-CD26 (Novus Biologicals) and rabbit anti-FAP (Novus Biologicals)) were incubated overnight after a 1 hour-TBS-2% milk blockade. Alexa-fluor 594 and 488 were used as secondary antibodies for primary mouse and rabbit antibodies respectively (Invitrogen).

### Immunohistochemistry

Fixed adipose tissue portions were embedded in paraffin immediately upon reception. Protocols for immunochemistry have been described in [16] without the antigen retrieval step. We used mouse anti-CD26 1/100 (Novus Biologicals) and rabbit anti-FAP 1/50 (Novus Biologicals) prior to streptavidin-biotin detection (Covance Research Products). Experiments were performed with OM and SC adipose tissues and similar results were obtained. Negative controls were performed with normal mouse and rabbit serum.

### DPP4 inhibitor treatment

Mature adipocytes isolated from SC adipose tissues of 13 patients were seeded into 6-well plates for ceiling culture. At day 4 of ceiling culture, slides with adherent adipocytes were reversed into a new plate with 2ml DMEM-20% serum in each well. Cells were treated with 50μM DPP4 inhibitor (Millipore, St.Charles, MO) or vehicle for 24 hours. Three replicates were cultivated per condition and pooled together at day 5 into Qiazol buffer for RNA extraction and RT-PCR quantification. A dose response experiment was performed with 50, 100 and 150μM inhibitor concentrations. Isolated mature adipocytes from 11 tissues were cultivated in ceiling culture and treated twice with the inhibitor, at day 4 and day 5, they were then harvested at day 6 in Qiazol buffer for RNA extraction.

### Statistical Analyses

Statistical analyses were performed using JMP software. Time effects on lipid droplet size and C/EBPα expression levels according to DPP4 inhibitor doses were analyzed by MANOVA-repeated measures analysis [17]. Messenger RNA expression, as well as depot and sex differences were compared by matched pair t-test analysis.

## Results

### 1) Mature adipocytes from OM and SC adipose tissues can be dedifferentiated independently of subject characteristics

**Fig 1:**
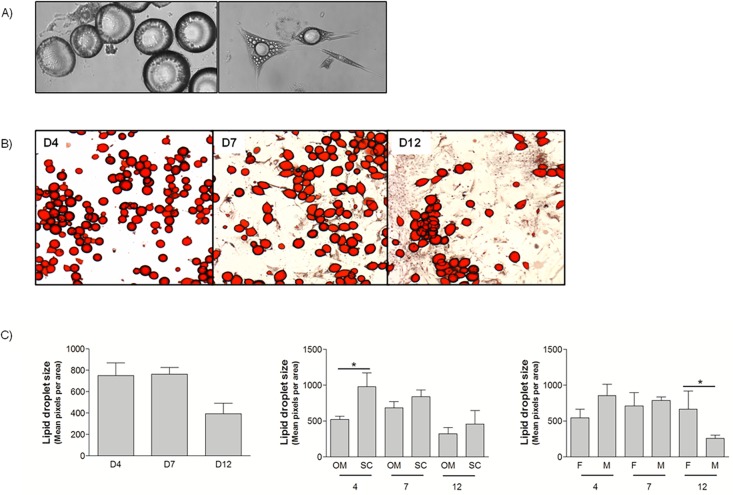
Dedifferentiating human mature adipocytes. A) Confocal microscopy of human mature adipocytes undergoing dedifferentiation at various stages (10x) B) Oil red O staining of OM mature adipocytes undergoing dedifferentiation in ceiling culture (4x). Days 4, 7 and 12 are shown. C) Lipid droplet size measurement of dedifferentiating mature adipocyte cells at days 4, 7 and 12 (n = 6, 4M; 2F, BMI range: 39–56.9 kg/m^2^, Age range: 29–59 years). Left panel: Statistical MANOVA analysis: Time p<0.05. Middle and right panels: Student T-tests *p<0.05.

We dedifferentiated collagenase-isolated mature adipocytes from the OM and SC fat depot obtained in obese men and women undergoing bariatric surgery. Using the same culture conditions, cells from 28 patients were successfully dedifferentiated for the purposes of this article. Patient characteristics are presented in Table 1. The cellular morphology of dedifferentiating adipocytes is clearly illustrated in Fig. 1A, where adherent round cells (left) and cells in dedifferentiation with elongated shapes and multiple droplets (right) can be observed. Fig. 1B shows representative OM mature adipocytes stained with ORO at days 4 and 7 of ceiling culture and at day 12 after initiating culture (7 days in ceiling plus 5 days in standard culture). At day 4, cells were round and attached to the support. The cell population was heterogeneous at day 7 with round cells and elongated cells. At day 12, some round cells remained, but most were fibroblast-like cells. Similar results were obtained with SC adipocytes. We maintained 10 different cultures longer than 12 days (paired OM and SC from 5 subjects, 4F;1M, age range from 36 to 66 years, BMI range from 40.9 to 71.8 kg/m^2^) to assess their capacity to proliferate in standard culture conditions. SC cultures reached an average of 16.2 passages (range: 13–24) and OM cultures reached an average of 15.8 passages (range: 13–21) without any apparent decrease in proliferative capacity. Cultures were stopped prior to senescence.

**Table 1:** Characteristics of the patients.

Characteristics	Mean ± SD	Min-Max
Age (years)	45.0 ± 12.6	23–62
Weight (kg)	136.1 ± 29.0	91.9–212.0
BMI (kg/m^2^)	48.5± 7.2	35.5–64.0
Comorbidities	Yes : No	
Diabetes	18 : 5	
Hypertension	13 : 11	
Dyslipidemia	8 : 16	
Sex (F : M)	13 : 11	

From ORO-stained cells, we counted red pixels to assess lipid droplet size in cultures from 6 subjects. Lipid droplet size was statistically modulated by time (p<0.05). When separated by depot, lipid droplet size was only significantly lower at day 4 with SC cells being larger than OM cells at that time point. We also evaluated differences between men and women and found significantly smaller lipid droplets in men vs women at day 12. These results also demonstrate successful dedifferentiation of mature adipocytes isolated from OM and SC adipose tissues of morbidly obese men and women.

### 2) Secretion of cytokines during dedifferentiation

**Fig 2:**
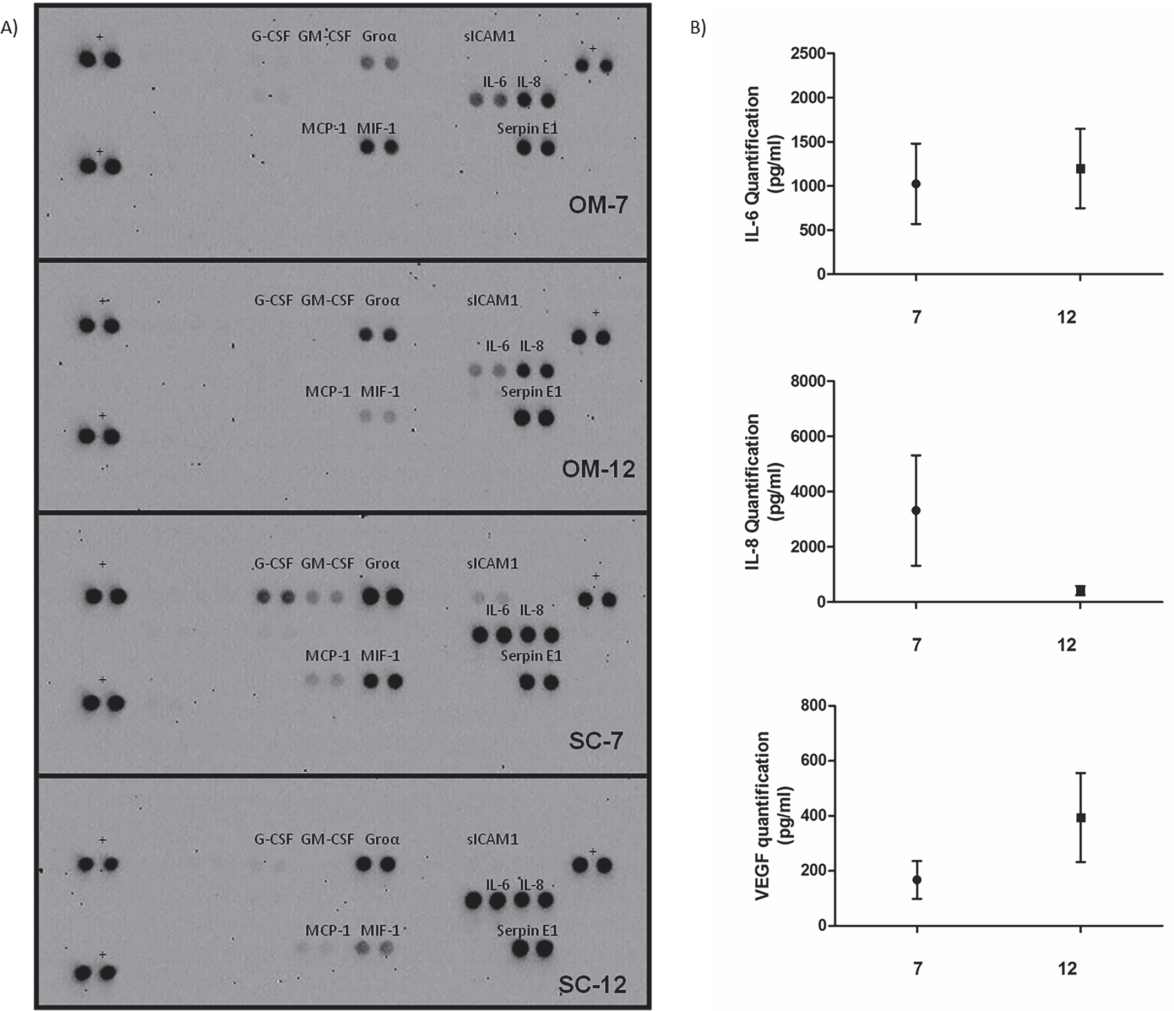
Cytokines secreted by dedifferentiating OM and SC mature adipocytes. A) Culture media from OM and SC adipocytes in dedifferentiation were blotted on cytokine membranes (representative results shown). Positive cytokines appear as black dots, in duplicate. +: membrane positive control B) Graphs of IL-6 (top panel), IL-8 (middle panel) and VEGF (bottom panel) values measured by Bioplex in media of dedifferentiating adipocytes at days 7 and 12.

We examined cytokine secretion during dedifferentiation with cytokine arrays and bio-plex at days 7 and 12. We found that IL-6, IL-8, Groα and Serpin E1 secretion was increased in dedifferentiating cells in all tested cultures with OM and SC cells (Fig. 2A). As shown on Fig. 2B, Bioplex analysis revealed high secretion levels of IL-6 and IL-8 as well as VEGF during the process. The analysis showed that IL-6 secretion remained constant between day 7 and day 12, whereas IL-8 secretion decreased and VEGF secretion is increased (Fig. 2B). Serpin E1 and Groα were not included in the Bioplex analysis. Our array revealed staining of MCP-1, G-CSF and GM-CSF only in SC media, but these cytokines were detected in OM media with the Bioplex assay. MIF-1 was detected mostly at day 7 compared to day 12 on the array (Fig. 2A). Table 2 indicates other cytokines not detected or with low or variable levels in the media tested in the bioplex and in the array.

**Table 2:** Cytokine detection in dedifferentiating mature adipocyte culture media by bioplex analysis.

Secretion	Cytokines (Bioplex)
Negative	IL-1β, IL-2, IL-4, IL-5, IL-7, IL-9, IL-10, IL-13, IL-15, IL-17, eotaxin, TNFα, Rantes, MIP-1β, PDGF-bb
Positive	IL-6, IL-8, G-CSF, GM-CSF, MCP-1, VEGF
Variable or low	IL-1ra, IL-12, IFNγ, FGF basic, MIP-1a
Secretion	Cytokines (Arrays)
Negative	C5a, CD40 ligand, CCL1, IFNγ, IL-1α, IL-1β, IL-1ra, IL-2, IL-4, IL-5, IL-10, IL-12, IL-13, IL-16, IL-17, IL-17E, IL-23, IL-27, IL-32α, CXCL10, CXCL11, MIP-1α, MIP-1β, Rantes, CXCL12, TNFα, TREM-1

### 3) Mature adipocyte gene expression rapidly decreases during ceiling culture for both depots

**Fig 3:**
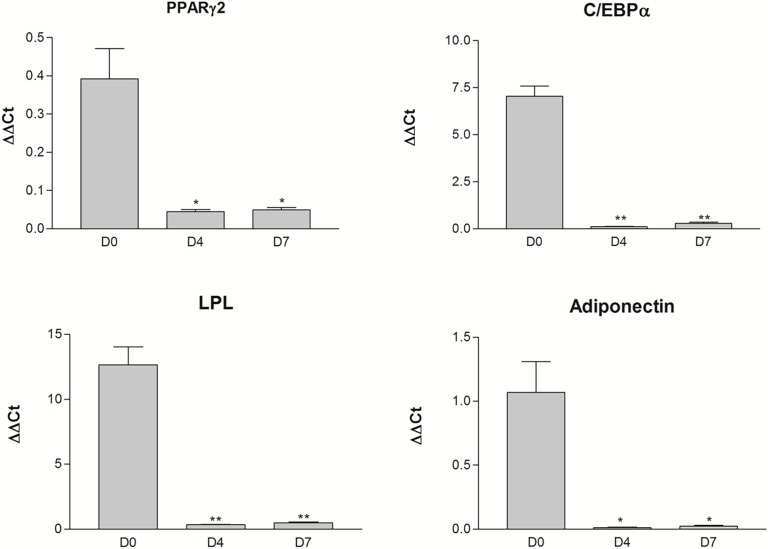
Expression levels of transcripts associated with mature adipocyte functions over a dedifferentiation time-course. PPARγ2, C/EBPα, LPL and Adiponectin expression levels were measured in mature adipocytes (D0) and in dedifferentiating adipocytes at days 4 and 7 (D4-D7) of ceiling culture. Data are expressed as ΔΔCt relative to G6PD expression (Mean value ± SEM, n = 4). Student t-test * p≤0.01, ** p<0.001.

We investigated four terminal markers of adipocyte function (PPARγ2, C/EBPα, LPL and Adiponectin) over a time course of dedifferentiation. Cells at day 0 correspond to mature adipocytes isolated by collagenase digestion of adipose tissue and cells at other time points, days 4 and 7, were obtained from ceiling culture. All four transcripts were expressed in adipose tissues but were absent from preadipocytes of the stromal-vascular fraction (data not shown). Accordingly, these transcripts were strongly expressed in mature adipocytes (day 0) as shown on Fig. 3. They were drastically down-regulated as early as four days in ceiling culture (Fig. 3), when most cells still had the cellular morphology of lipid-storing adipocytes (Fig. 1A). Matched pair t-tests indicated statistically significant differences in gene expression between day 0 and days 4 or 7 (p<0.05) for all genes. Gene expression remained below detection limits for every sample at day 12, which was similar to the expression observed in paired preadipocytes (data not shown). We did not find any significant depot difference in gene expression for these transcripts. Genes related to lipid storage and mature adipocyte function were down-regulated early during dedifferentiation, independently of cellular morphology and cells from both fat compartments exhibited similar expression. Moreover, DFAT cells were similar to preadipocytes based on expression of these transcripts.

### 4) Matrix remodeling genes are strongly upregulated in dedifferentiating adipocytes

**Fig 4:**
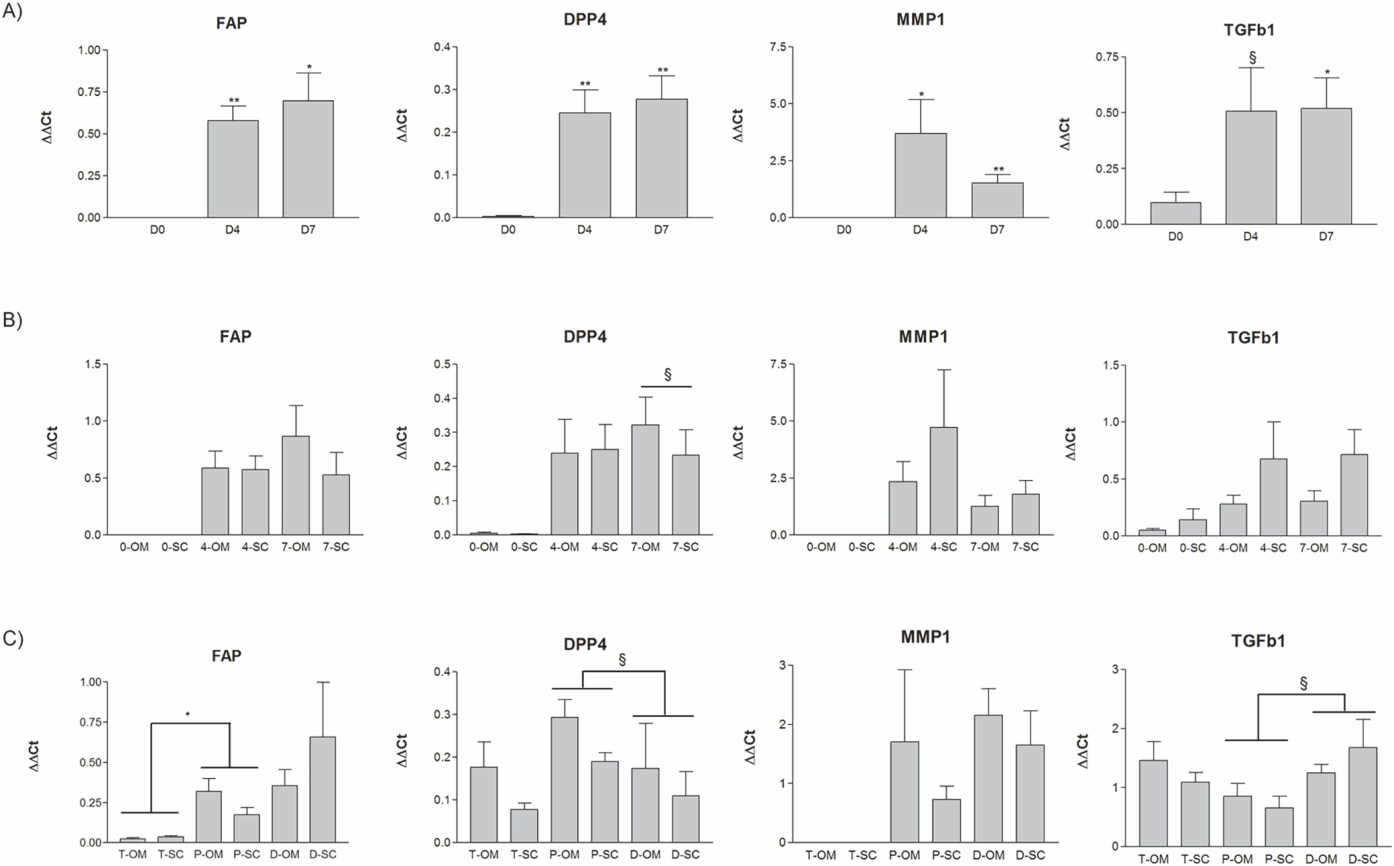
Relative gene expression of matrix remodeling genes in dedifferentiating adipocytes. A) FAP, DPP4, MMP1 and TGFβ1 expression levels were measured in mature adipocytes (D0) and in dedifferentiating adipocytes at days 4 and 7 (D4-D7) of ceiling culture. B) Data are expressed with depot-specific expression at days 0, 4 and 7 for omental (OM) and subcutaneous (SC) cells. C) Comparison of gene expression between adipose tissues, preadipocytes and DFAT cells from the OM and SC fat depots. Data are expressed as mean value of ΔΔCt relative to G6PD expression ± SEM (n = 4). Matched pair t-test: *p<0.05, ** p<0.01, § p<0.10.

As previously mentioned, dedifferentiation led to profound changes in cell morphology which was strongly related to modulation of matrix remodeling gene expression. We found that MMP1, FAP, DPP4 and TGFβ1 expression levels were strongly induced during dedifferentiation as shown on Fig. 4A. These genes were expressed in low to undetectable levels in isolated mature adipocytes (day 0) and expression levels were strongly increased at days 4 and 7 of ceiling culture, irrespective of depot source. Matched pair t-tests were performed to compare expression between day 0 and days 4 or 7 and differences were statistically significant, except for TGFβ1 for which a trend between days 0 and 4 was observed (p = 0.105). Fig. 4B illustrates depot-specific expression of genes at days 0, 4, and 7. We did not find any statistically significant difference in gene expression between OM and SC at these time points. We then compared expression of FAP, DPP4, MMP1 and TGFβ1 in OM and SC adipose tissues, preadipocytes and DFAT cells. Except for MMP1, all genes were expressed in adipose tissues and all four were expressed in preadipocytes. Fig. 4C shows expression in DFAT cells at day 12. We did not find statistically significant differences between the OM and SC fat depots. Moreover, DPP4 tended to have lower expression levels in DFAT compared to preadipocytes (p = 0.09) whereas TGFβ1 tended to be more highly expressed in DFAT (p = 0.06).

### 5) FAP and DPP4 proteins are induced during mature adipocyte dedifferentiation and are detected in human adipose tissues

**Fig 5:**
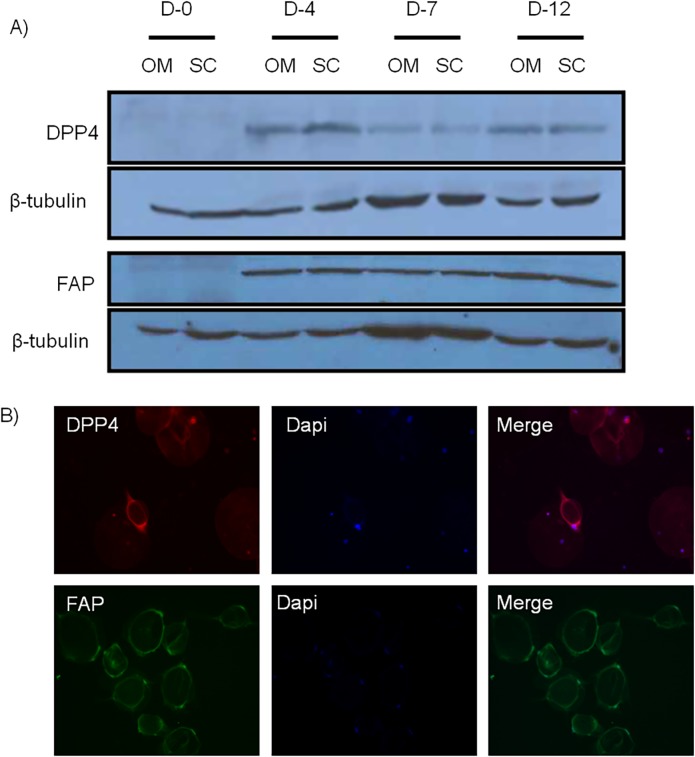
FAP and DPP4 protein expression in mature adipocytes and whole adipose tissue. A) FAP and DPP4 proteins are induced during adipocyte dedifferentiation in both OM and SC fat depots at various time-points. β-tubulin was used as loading control. B) Immunofluorescent FAP and DPP4 proteins were detected in 4 days-dedifferentiating mature adipocytes. (D: day, OM: omental, SC: subcutaneous)

FAP and DPP4 are particularly interesting because of their protein similarity and functions. We first measured their protein levels in adipocytes undergoing dedifferentiation. As shown on Fig. 5A, FAP and DPP4 proteins were not detected in mature adipocytes before dedifferentiation, but both were strongly induced by dedifferentiation as observed in samples collected at days 4, 7 and 12. Both depots expressed similar amounts of protein which is consistent with mRNA levels. Protein expression was also shown by immunofluorescence imaging of DFAT cells at day 4 of ceiling culture (Fig. 5B). Immunofluorescent proteins were also detected in cells at days 7 and 12 (data not shown).

**Fig 6:**
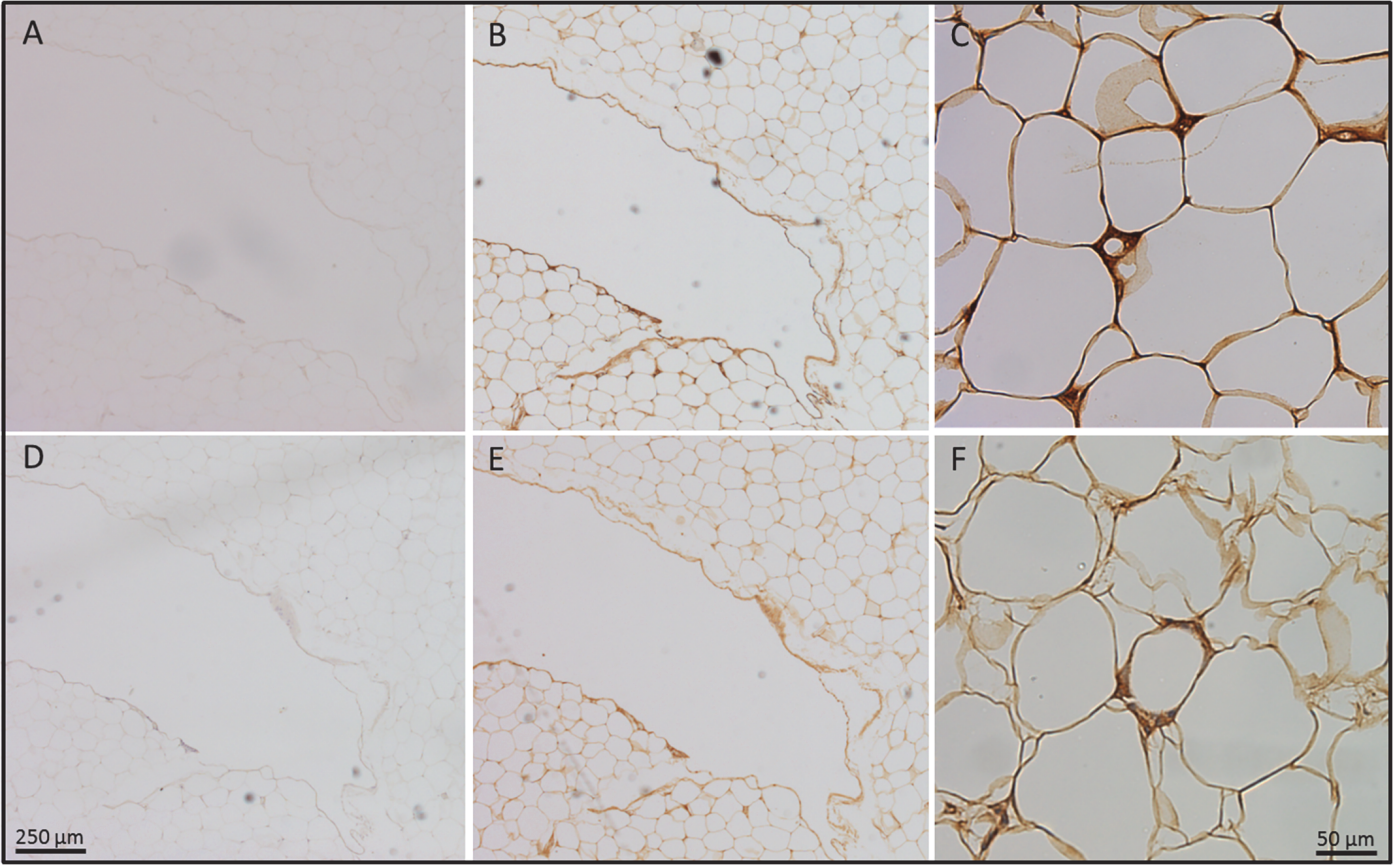
FAP and DPP4 protein detection in whole adipose tissue by immunohistochemistry from a 59-year old man with a BMI of 35.5 kg/m^2^. A) Negative control-mouse antiserum, B) DPP4 antibody 4x, C) DPP4 antibody 20x, E) Negative control-rabbit antiserum, F) FAP antibody 4x, G) FAP antibody 20x. Scale bar: 250 μM for panels A, B, D and E, 50 μM for C and F.

We examined protein expression of FAP and DPP4 in obese adipose tissue depots by immunohistochemistry. As shown in Fig. 6, both DPP4 (Fig. 6B and C) and FAP (Fig. e and f) were detected in the intercellular spaces, that may correspond to the stromal-vascular fraction (SVF) of adipose tissue. In comparison to FAP, we observed a strong DPP4 staining in mature adipocytes, possibly localized at the cell membrane. This is concordant with the qPCR results (Fig. 4C) where FAP was mostly detected in the SVF compared to whole adipose tissue samples. DPP4 was more abundant in whole adipose tissue than FAP.

### 6) Treatment of dedifferentiating adipocytes with a DPP4 inhibitor affected mRNA expression of C/EBPα, PPARγ2 and Adiponectin

**Fig 7:**
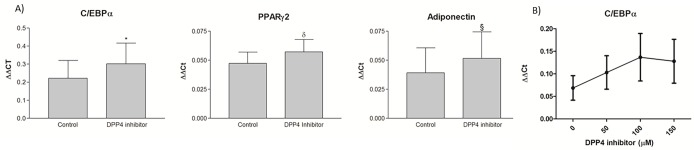
Effects of a DPP4 inhibitor on gene expression of dedifferentiating SC adipocytes. A) C/EBPα, PPARγ2 and adiponectin gene expression were quantified in mature adipocytes undergoing dedifferentiation (day 5) after a 24-hour treatment with a DPP4 inhibitor (50 μM) or vehicle (Control). N = 13 patients, Matched pair t-test: *p<0.05, § p≤0.10, δ p≤0.15. B) Dose response of DPP4 inhibitor on C/EBPα expression levels in dedifferentiating adipocytes. Cells were treated at days 4 and 6 of ceiling culture in a 6-well plate and harvested at day 6 for qPCR analysis. Repeated measures analysis p-value = 0.06.

In order to verify the impact of DPP4 during mature adipocyte dedifferentiation, we treated cells for 24 hours with or without a DPP4 inhibitor (Millipore) at a final concentration of 50μM. We compared gene expression related to mature adipocyte functions, which is lost during dedifferentiation at day 5. We found that the DPP4 inhibitor slightly attenuated the down-regulation of genes associated to the mature adipocyte phenotype. C/EBPα expression was significantly affected by the inhibitor (p = 0.05), whereas the effect on adiponectin mRNA expression tended to be significant (p = 0.08). PPARγ2 expression was also close to trending (p = 0.15). We performed a dose response with the DPP4 inhibitor and examined mRNA expression levels of C/EBPα. As shown in Fig. 7B, the increasing doses of inhibitor increased levels of C/EBPα (MANOVA dose effect p = 0.06), which suggests that the inhibitor blunted the decrease of C/EBPα expression observed during dedifferentiation. Although modest in the conditions tested, these results suggest an effect of DPP4 inhibition on adipogenic gene expression through the process of dedifferentiation.

## Discussion

We present our work on the characterization of mature adipocytes undergoing dedifferentiation at various time points with a particular attention to the OM and SC abdominal fat compartments. First, we demonstrated that cells from all the tested patients can undergo the dedifferentiation process. We obtained cells from morbidly obese male and female patients undergoing bariatric surgery and ranging in age from 29 to 66 years and we successfully dedifferentiated fat cells from the OM and SC abdominal compartments. Matsumoto and collaborators had previously obtained adipose samples from the SC fat compartments of 18 donors with a wide age range. Poloni et al. had recruited 12 non-obese patients between 53 and 81 years old who had undergone adipose tissue sampling from the OM and SC depots. They had observed no difference between OM and SC DFAT cells, but they had examined fully dedifferentiated cells. Our data presenting depot differences in the size of lipid droplets at day 4 and sex differences at day 12 suggest that cellular dedifferentiation capacities could vary quantitatively, but not qualitatively between depots and among individuals. The effect of the sex of the donor on dedifferentiation efficiency has never been reported in the literature. But, as a dimorphism is observed in body fat distribution, intrinsic differences in cell behaviour could be explained by hormonal responses. Androgens are known to inhibit preadipocyte differentiation of cells originating from men and women [18]. We found that men had smaller lipid droplets at day 12. This may suggest greater dedifferentiation for cells originating from a more androgenic environment. The inhibitory effect of androgens in differentiation could possibly represent a stimulatory effect for dedifferentiation. However, additional studies are needed to test hormonal effects on this process.

We did not specifically control our sampling for the degree of obesity and metabolic dysfunction, but our patients were all severely obese and the majority of them were characterized by type 2 diabetes. Our ability to dedifferentiate adipocytes from these patients suggests that cells from an obese and possibly metabolically dysfunctional environment can still undergo dedifferentiation. Poloni and collaborators demonstrated the viability of mature adipocytes in ceiling cultures showing BrDU incorporation in these cells during dedifferentiation. Adipocyte viability and the replicative capacity of fully dedifferentiated cells were not the main focus of this study. However, OM and SC DFAT cells have been cultivated in our laboratory for more than 12 passages, some until 23–24 passages (n = 6 per depot), suggesting a high replicative capacity and viability. These cells were isolated from adipose tissues of obese women aged between 36 and 66 years. Regarding the effects of age, Matsumoto et al. observed a decreasing replicative capacity of DFAT cells from patients older than 70 years [3]. The efficiency of dedifferentiation was not evaluated in this study as we are still developing tools to do so. Here, we assessed capacity of cells to dedifferentiate by lipid droplet size analysis. Irrespective of age, we have successfully induced dedifferentiation in all ceiling cultures. More studies are needed to evaluate how patient characteristics may influence the efficiency of the process.

We are the first group to investigate dedifferentiation as a physiological process instead of focusing exclusively on the final resulting cells. Cellular morphology is profoundly affected by the process over time, and we demonstrated that gene expression variation occurs early during dedifferentiation. In fact, although many cells are still round at day 4, their cellular programs are profoundly modified, especially for transcripts encoding proteins involved in mature adipocyte functions or matrix remodeling. We show down-regulation of mature adipocyte transcripts (PPARγ, C/EBPα, LPL and ADIPOQ). Some of these genes (PPARγ and C/EBPα) have been shown to be downregulated in DFAT cells of human and porcine origins, but these data were only obtained from completely dedifferentiated cells [3,8,13]. Here, we report an early down-regulation, indicating that cells may not need to be completely transformed to change this cellular program.

Adipose tissue remodeling is a recently-investigated aspect of the pathophysiology of obesity and efforts have been made to better understand this phenomenon [19]. Ono et al. reported that genes implicated in cell morphology, movement and other pathways of cellular modification were different between porcine adipocytes and DFAT cells; this supports the notion of cellular plasticity in adipocytes. In this report we show that matrix remodeling genes are strongly induced during dedifferentiation *in vitro*. We can only speculate as to whether dedifferentiation is involved in adipose tissue remodeling in the context of obesity. It could be an adaptive mechanism to modulate lipid storage capacity in conditions of weight gain or weight loss by increasing or decreasing the number of cells available. More studies will be necessary to identify the inducer of mature adipocyte dedifferentiation. Cellular plasticity, including the dedifferentiation phenomenon, has been described in other pathologies. For example, β-cells have been shown to dedifferentiate following metabolic stress in mice [20]. *In vitro* studies also demonstrated dedifferentiation of thyroid cells with endoplasmic reticulum stress [21] and retinal glial cells after an oxidative stress [22] supporting the adaptive hypothesis.

The *in vitro* inducer of dedifferentiation is still unknown. Several hypotheses have been put forward such as gas exchange, high-serum condition and cell-plastic contact. Our 6-well plate model allows for gas exchanges, which may rule out a predominant effect of hypoxia. High-serum conditions may be an important aspect of the *in vitro* culture, but we tested lower serum concentrations and still observed cell adherence and dedifferentiation (data not shown). The cell-plastic contact is probably an important aspect of the process, especially with the observed increase in expression of genes related to matrix remodelling.

Matrix metalloproteinases (MMPs) are a class of proteins known to participate in matrix remodeling. In addition to our discovery of MMP1 upregulation during dedifferentiation, MMP3 has been recently reported to be up-regulated in DFAT cells compared to adipose-derived stem cells [10]. Interestingly, MMP1 has been reported as the most up-regulated MMP in adipocytes cultivated with macrophage-conditioned medium and has been shown to be secreted in high amounts by these cells [23]. In the same study, IL-6 and IL-8 mRNA levels were also remarkably increased in adipocytes exposed to macrophage-conditioned medium [23]. The relation between MMP1 expression and secretion in preadipocytes or adipocytes and inflammation was demonstrated by Gao et al. [24]. In addition, some MMPs and their inhibitors (TIMP) have been reported to be modulated with obesity levels in mice and humans, supporting their possible implication in this pathophysiology [25–27]. Because MMP-2 and MMP-9 are essential to adipocyte differentiation, we propose that other MMPs could be involved in reversing this process.

TGFβ1 has also been reported as an important player in adipocyte physiology. Some studies have demonstrated that TGFβ1 inhibits adipogenesis [28,29]. PAI-1 (or SerpinE1) is a downstream target of TGFβ1. Interestingly, PAI-1 is secreted in high amounts by mature adipocytes undergoing dedifferentiation and also expressing high levels of TGFβ1. Moreover, TGFβ1 has frequently been described as an important factor in matrix remodeling and cell motility in several pathological and physiological conditions (reviewed in [30]). Our results suggest that, similar to MMP1, TGFβ1 and Serpin E1 could be involved in reversing the cellular adipocyte phenotype.

We are the first to describe a putative function for FAP in adipose tissue biology. FAP has mainly been found in pathological environments (fibrosis, keloids, cancer and arthritis) [31–34]. This protein is a transmembrane serine peptidase from the prolyl peptidase family which also comprises as a member DPP4 (also known as CD26) [34]. FAP and DPP4 have the highest homology among members of this family and they can form heterodimers [35]. Their coexpression has been previously described in other cell types such as astrocytes, where they influence cancer invasiveness [36]. FAP and DPP4 modulate extracellular matrix degradation by cleaving collagen molecules for tissue remodeling purposes in various contexts [34,37]. Moreover, FAP and MMP could be working together in some cellular environments, as demonstrated by Huang and collaborators in breast cancer cells [38]. In addition, FAP could be an indirect target of the TGFβ signaling pathway in breast cancer [34]. Together with the strong induction of FAP and DPP4 in dedifferentiating fat cells, available data suggest that these proteins may participate in matrix remodeling of adipocytes undergoing dedifferentiation.

DPP4 has been linked to obesity and diabetes [39–41]. DPP4 inhibitors are used as oral hypoglycemic agents and their role is based on incretin activity modulation and subsequent increases in insulin secretion [42]. Many recent publications have linked DPP4 with the physiopathology of obesity. First, DPP4 expression was reported to be higher in visceral adipose tissue of obese subjects compared to SC tissue [39]. In addition, DPP4 release by adipose tissue was correlated with adipocyte size and features of the metabolic syndrome [39]. Bouchard et al. found overexpression of DPP4 in OM adipose tissue in men with the metabolic syndrome compared to obese controls without metabolic alterations [43]. Also, methylation rates of the DPP4 gene in adipose tissue were associated with plasma lipid concentrations in obese men [41,44]. Through its action on NPY, DPP4 has been reported to be involved in the regulation of adipose tissue lipolysis [45]. A possible effect of DPP4 in adipose tissue physiology is also supported by a study from Dobrian et al. demonstrating a change in adipose cell composition in mice following DPP4 inhibitor sitagliptin treatment [46]. The demonstration that a DPP4 inhibitor may affect gene expression of mature cells during dedifferentiation indirectly supports the idea that it may play a role in adipocyte dedifferentiation. The modest effect in some cultures may be due to a compensatory mechanism by MMP and/or other remodelling proteins or due to inter-individual variations. Emerging evidence, combined with our findings, supports the hypothesis that DPP4 could be a significant protein for adipose tissue remodeling with possible impact on obesity and metabolic diseases.

We examined cytokine secretion during dedifferentiation. Two studies had performed bioplex analyses, but had used media from fully dedifferentiated cells [8,10]. The main cytokines detected in our media by bioplex during dedifferentiation are generally similar to those previously identified in DFAT cells. Secretion remains high for some cytokines (IL-6, IL-8 and VEGF) suggesting that they may play a role throughout the process. Inflammation is part of the physiopathology of obesity and high levels of pro-inflammatory cytokines such as IL6, IL-8 and Serpin E1 could link together inflammation and adipose tissue remodeling. Accordingly, Macrophage Migration Inhibitory Factor (MIF) expression was higher at day 7 than 12, so it could be more important in the middle of the process than other cytokines examined. In addition, most secreted cytokines were common to all subjects tested whereas those secreted at lower levels showed more variability suggesting possible inter-individual differences. Once again, intrinsic characteristics of the donors may influence this aspect of the process.

## Conclusion

In this study of adipocyte dedifferentiation, we report considerable changes in cytokine secretion and gene expression early during the *in vitro* cellular process. More precisely, matrix remodeling transcripts (FAP, DPP4, MMP1 and TGFβ1) are rapidly and strongly up-regulated through the process. Our findings are consistent with a role for FAP and DPP4 in adipose tissue remodeling and cell plasticity.

## References

[1] Cinti S (2012) The adipose organ at a glance. Dis Model Mech 5: 588–594. 10.1242/dmm.009662 22915020 PMC3424455

[2] Lowe CE, O'Rahilly S, Rochford JJ (2011) Adipogenesis at a glance. J Cell Sci 124: 2681–2686. 10.1242/jcs.079699 21807935

[3] Matsumoto T, Kano K, Kondo D, Fukuda N, Iribe Y, et al (2008) Mature adipocyte-derived dedifferentiated fat cells exhibit multilineage potential. J Cell Physiol 215: 210–222. 18064604 10.1002/jcp.21304

[4] Frontini A, Vitali A, Perugini J, Murano I, Romiti C, et al (2013) White-to-brown transdifferentiation of omental adipocytes in patients affected by pheochromocytoma. Biochim Biophys Acta 1831: 950–959. 10.1016/j.bbalip.2013.02.005 23454374

[5] Ohno H, Shinoda K, Spiegelman BM, Kajimura S (2012) PPARgamma agonists induce a white-to-brown fat conversion through stabilization of PRDM16 protein. Cell Metab 15: 395–404. 10.1016/j.cmet.2012.01.019 22405074 PMC3410936

[6] Zhang HH, Kumar S, Barnett AH, Eggo MC (2000) Ceiling culture of mature human adipocytes: use in studies of adipocyte functions. J Endocrinol 164: 119–128. 10657847 10.1677/joe.0.1640119

[7] Nobusue H, Kano K (2010) Establishment and characteristics of porcine preadipocyte cell lines derived from mature adipocytes. J Cell Biochem 109: 542–552. 10.1002/jcb.22431 20013788

[8] Poloni A, Maurizi G, Leoni P, Serrani F, Mancini S, et al (2012) Human dedifferentiated adipocytes show similar properties to bone marrow-derived mesenchymal stem cells. Stem Cells 30: 965–974. 10.1002/stem.1067 22367678

[9] Nobusue H, Endo T, Kano K (2008) Establishment of a preadipocyte cell line derived from mature adipocytes of GFP transgenic mice and formation of adipose tissue. Cell Tissue Res 332: 435–446. 10.1007/s00441-008-0593-9 18386066

[10] Perrini S, Ficarella R, Picardi E, Cignarelli A, Barbaro M, et al (2013) Differences in gene expression and cytokine release profiles highlight the heterogeneity of distinct subsets of adipose tissue-derived stem cells in the subcutaneous and visceral adipose tissue in humans. PLoS One 8: e57892 10.1371/journal.pone.0057892 23526958 PMC3589487

[11] Gao Q, Zhao L, Song Z, Yang G (2012) Expression pattern of embryonic stem cell markers in DFAT cells and ADSCs. Mol Biol Rep 39: 5791–5804. 10.1007/s11033-011-1371-4 22237862

[12] Shen JF, Sugawara A, Yamashita J, Ogura H, Sato S (2011) Dedifferentiated fat cells: an alternative source of adult multipotent cells from the adipose tissues. Int J Oral Sci 3: 117–124. 10.4248/IJOS11044 21789960 PMC3470092

[13] Ono H, Oki Y, Bono H, Kano K (2011) Gene expression profiling in multipotent DFAT cells derived from mature adipocytes. Biochem Biophys Res Commun 407: 562–567. 10.1016/j.bbrc.2011.03.063 21419102

[14] Blouin K, Richard C, Belanger C, Dupont P, Daris M, et al (2003) Local androgen inactivation in abdominal visceral adipose tissue. J Clin Endocrinol Metab 88: 5944–5950. 14671194 10.1210/jc.2003-030535

[15] Koopman R, Schaart G, Hesselink MK (2001) Optimisation of oil red O staining permits combination with immunofluorescence and automated quantification of lipids. Histochem Cell Biol 116: 63–68. 11479724 10.1007/s004180100297

[16] Poisson Pare D, Song D, Luu-The V, Han B, Li S, et al (2009) Expression of Estrogen Sulfotransferase 1E1 and Steroid Sulfatase in Breast Cancer: A Immunohistochemical Study. Breast Cancer (Auckl) 3: 9–21. 21556246 10.4137/bcbcr.s2012 PMC3086308

[17] O'Brien RG, Kaiser MK (1985) MANOVA method for analyzing repeated measures designs: an extensive primer. Psychol Bull 97: 316–333. 3983301

[18] Blouin K, Nadeau M, Perreault M, Veilleux A, Drolet R, et al (2010) Effects of androgens on adipocyte differentiation and adipose tissue explant metabolism in men and women. Clin Endocrinol (Oxf) 72: 176–188. 10.1111/j.1365-2265.2009.03645.x 19500113

[19] Lee MJ, Wu Y, Fried SK (2010) Adipose tissue remodeling in pathophysiology of obesity. Curr Opin Clin Nutr Metab Care 13: 371–376. 10.1097/MCO.0b013e32833aabef 20531178 PMC3235038

[20] Talchai C, Xuan S, Lin HV, Sussel L, Accili D (2012) Pancreatic beta cell dedifferentiation as a mechanism of diabetic beta cell failure. Cell 150: 1223–1234. 10.1016/j.cell.2012.07.029 22980982 PMC3445031

[21] Abrahan CE, Insua MF, Politi LE, German OL, Rotstein NP (2009) Oxidative stress promotes proliferation and dedifferentiation of retina glial cells in vitro. J Neurosci Res 87: 964–977. 10.1002/jnr.21903 18855938

[22] Ulianich L, Garbi C, Treglia AS, Punzi D, Miele C, et al (2008) ER stress is associated with dedifferentiation and an epithelial-to-mesenchymal transition-like phenotype in PC Cl3 thyroid cells. J Cell Sci 121: 477–486. 10.1242/jcs.017202 18211961

[23] O'Hara A, Lim FL, Mazzatti DJ, Trayhurn P (2009) Microarray analysis identifies matrix metalloproteinases (MMPs) as key genes whose expression is up-regulated in human adipocytes by macrophage-conditioned medium. Pflugers Arch 458: 1103–1114. 10.1007/s00424-009-0693-8 19585142

[24] Gao D, Bing C (2011) Macrophage-induced expression and release of matrix metalloproteinase 1 and 3 by human preadipocytes is mediated by IL-1beta via activation of MAPK signaling. J Cell Physiol 226: 2869–2880. 10.1002/jcp.22630 21935932

[25] Chavey C, Mari B, Monthouel MN, Bonnafous S, Anglard P, et al (2003) Matrix metalloproteinases are differentially expressed in adipose tissue during obesity and modulate adipocyte differentiation. J Biol Chem 278: 11888–11896. 12529376 10.1074/jbc.M209196200

[26] Papazoglou D, Papatheodorou K, Papanas N, Papadopoulos T, Gioka T, et al (2010) Matrix metalloproteinase-1 and tissue inhibitor of metalloproteinases-1 levels in severely obese patients: what is the effect of weight loss? Exp Clin Endocrinol Diabetes 118: 730–734. 10.1055/s-0030-1249671 20361393

[27] Maquoi E, Munaut C, Colige A, Collen D, Lijnen HR (2002) Modulation of adipose tissue expression of murine matrix metalloproteinases and their tissue inhibitors with obesity. Diabetes 51: 1093–1101. 11916931 10.2337/diabetes.51.4.1093

[28] Choy L, Derynck R (2003) Transforming growth factor-beta inhibits adipocyte differentiation by Smad3 interacting with CCAAT/enhancer-binding protein (C/EBP) and repressing C/EBP transactivation function. J Biol Chem 278: 9609–9619. 12524424 10.1074/jbc.M212259200

[29] Choy L, Skillington J, Derynck R (2000) Roles of autocrine TGF-beta receptor and Smad signaling in adipocyte differentiation. J Cell Biol 149: 667–682. 10791980 10.1083/jcb.149.3.667 PMC2174852

[30] Gordon KJ, Blobe GC (2008) Role of transforming growth factor-beta superfamily signaling pathways in human disease. Biochim Biophys Acta 1782: 197–228. 10.1016/j.bbadis.2008.01.006 18313409

[31] Dienus K, Bayat A, Gilmore BF, Seifert O (2010) Increased expression of fibroblast activation protein-alpha in keloid fibroblasts: implications for development of a novel treatment option. Arch Dermatol Res 302: 725–731. 10.1007/s00403-010-1084-x 20872224

[32] Lee HO, Mullins SR, Franco-Barraza J, Valianou M, Cukierman E, et al (2011) FAP-overexpressing fibroblasts produce an extracellular matrix that enhances invasive velocity and directionality of pancreatic cancer cells. BMC Cancer 11: 245 10.1186/1471-2407-11-245 21668992 PMC3141768

[33] Lai D, Ma L, Wang F (2012) Fibroblast activation protein regulates tumor-associated fibroblasts and epithelial ovarian cancer cells. Int J Oncol 41: 541–550. 10.3892/ijo.2012.1475 22614695

[34] Kelly T, Huang Y, Simms AE, Mazur A (2012) Fibroblast activation protein-alpha: a key modulator of the microenvironment in multiple pathologies. Int Rev Cell Mol Biol 297: 83–116. 10.1016/B978-0-12-394308-8.00003-0 22608558

[35] Busek P, Malik R, Sedo A (2004) Dipeptidyl peptidase IV activity and/or structure homologues (DASH) and their substrates in cancer. Int J Biochem Cell Biol 36: 408–421. 14687920 10.1016/s1357-2725(03)00262-0

[36] Balaziova E, Busek P, Stremenova J, Sromova L, Krepela E, et al (2011) Coupled expression of dipeptidyl peptidase-IV and fibroblast activation protein-alpha in transformed astrocytic cells. Mol Cell Biochem 354: 283–289. 10.1007/s11010-011-0828-z 21526345

[37] Wang XM, Yu DM, McCaughan GW, Gorrell MD (2006) Extra-enzymatic roles of DPIV and FAP in cell adhesion and migration on collagen and fibronectin. Adv Exp Med Biol 575: 213–222. 16700525 10.1007/0-387-32824-6_23

[38] Huang Y, Simms AE, Mazur A, Wang S, Leon NR, et al (2011) Fibroblast activation protein-alpha promotes tumor growth and invasion of breast cancer cells through non-enzymatic functions. Clin Exp Metastasis 28: 567–579. 10.1007/s10585-011-9392-x 21604185

[39] Lamers D, Famulla S, Wronkowitz N, Hartwig S, Lehr S, et al (2011) Dipeptidyl peptidase 4 is a novel adipokine potentially linking obesity to the metabolic syndrome. Diabetes 60: 1917–1925. 10.2337/db10-1707 21593202 PMC3121429

[40] Blouin K, Despres JP, Couillard C, Tremblay A, Prud'homme D, et al (2005) Contribution of age and declining androgen levels to features of the metabolic syndrome in men. Metabolism 54: 1034–1040. 16092053 10.1016/j.metabol.2005.03.006

[41] Turcot V, Tchernof A, Deshaies Y, Perusse L, Belisle A, et al (2013) Comparison of the dipeptidyl peptidase-4 gene methylation levels between severely obese subjects with and without the metabolic syndrome. Diabetol Metab Syndr 5: 4 10.1186/1758-5996-5-4 23379505 PMC3637825

[42] Ross SA, Ekoe JM (2010) Incretin agents in type 2 diabetes. Can Fam Physician 56: 639–648. 20631270 PMC2922799

[43] Bouchard L, Faucher G, Tchernof A, Deshaies Y, Lebel S, et al (2009) Comprehensive genetic analysis of the dipeptidyl peptidase-4 gene and cardiovascular disease risk factors in obese individuals. Acta Diabetol 46: 13–21. 10.1007/s00592-008-0049-4 18682883

[44] Turcot V, Bouchard L, Faucher G, Tchernof A, Deshaies Y, et al (2011) DPP4 gene DNA methylation in the omentum is associated with its gene expression and plasma lipid profile in severe obesity. Obesity (Silver Spring) 19: 388–395. 10.1038/oby.2010.198 20847730

[45] Kos K, Baker AR, Jernas M, Harte AL, Clapham JC, et al (2009) DPP-IV inhibition enhances the antilipolytic action of NPY in human adipose tissue. Diabetes Obes Metab 11: 285–292. 10.1111/j.1463-1326.2008.00909.x 19175376

[46] Dobrian AD, Ma Q, Lindsay JW, Leone KA, Ma K, et al (2011) Dipeptidyl peptidase IV inhibitor sitagliptin reduces local inflammation in adipose tissue and in pancreatic islets of obese mice. Am J Physiol Endocrinol Metab 300: E410–421. 10.1152/ajpendo.00463.2010 21081706 PMC3043624

